# Primary Prevention of Food Allergy: Translating Evidence from Clinical Trials to Population-Based Recommendations

**DOI:** 10.1016/j.jaip.2017.12.015

**Published:** 2018

**Authors:** Paul J. Turner, Dianne E. Campbell, Robert J. Boyle, Michael E. Levin

**Affiliations:** a Section of Paediatrics (Allergy and Immunology), Imperial College London, London, UK; b Discipline of Child and Adolescent Health, University of Sydney, Sydney, Australia; c Department of Allergy and Immunology, Children's Hospital at Westmead, Sydney, Australia; d Division of Allergology, Department of Paediatrics, Faculty of Health Sciences, University of Cape Town, Cape Town, South Africa

**Keywords:** Allergy, Prevention, Peanut, Translation, Implementation, GRADE, Grading of Recommendations Assessment, Development and Evaluation, HIV, Human immunodeficiency virus, LEAP, Learning Early About Peanut, NNT, Number needed to treat, RCT, Randomized controlled trial, WAO, World Allergy Organization

## Abstract

Given the prevalence and impact of childhood food allergy, there is increasing interest in interventions targeting disease prevention. Although interventions such as early introduction of dietary peanut have demonstrated efficacy in a small number of well-conducted randomized clinical trials, evidence for broader effectiveness and successful implementation at a population level is still lacking, although epidemiological data suggest that such strategies are likely to be successful, at least for peanut. In this commentary, we explore the issues of translating evidence of efficacy studies (performed under optimal conditions) to make policy recommendations at a population level, and highlight potential benefits, harms, and unintended consequences of making population-based recommendations on the basis of randomized controlled trials. We discuss the complexity and barriers to effective primary and secondary prevention intervention implementation in resource-poor settings.

**Information for Category 1 CME Credit**

Credit can now be obtained, free for a limited time, by reading the review articles in this issue. Please note the following instructions.

**Method of Physician Participation in Learning Process:** The core material for these activities can be read in this issue of the Journal or online at the *JACI: In Practice* Web site: www.jaci-inpractice.org/. The accompanying tests may only be submitted online at www.jaci-inpractice.org/. Fax or other copies will not be accepted.

**Date of Original Release:** March 1, 2018. Credit may be obtained for these courses until February 28, 2019.

**Copyright Statement:** Copyright © 2018-2020. All rights reserved.

**Overall Purpose/Goal:** To provide excellent reviews on key aspects of allergic disease to those who research, treat, or manage allergic disease.

**Target Audience:** Physicians and researchers within the field of allergic disease.

**Accreditation/Provider Statements and Credit Designation:** The American Academy of Allergy, Asthma & Immunology (AAAAI) is accredited by the Accreditation Council for Continuing Medical Education (ACCME) to provide continuing medical education for physicians. The AAAAI designates this journal-based CME activity for 1.00 *AMA PRA Category 1 Credit*™. Physicians should claim only the credit commensurate with the extent of their participation in the activity.

**List of Design Committee Members:** Paul J. Turner, FRACP, PhD, Dianne E. Campbell, FRACP, PhD, Robert J. Boyle, MB ChB, PhD, and Michael E. Levin, MD (authors); Michael Schatz, MD, MS (editor)

**Learning objectives:**

1. To describe the issues in using study data to inform population-based interventions to prevent food allergy.
2. To understand the need to distinguish between Efficacy trials (which might reflect ideal circumstances) and Effectiveness trials (which assess effect in the “real world”).
3. To identify the current knowledge and evidence gaps in strategies that have been considered to potentially prevent food allergy at a population level.

**Recognition of Commercial Support:** This CME has not received external commercial support.

**Disclosure of Relevant Financial Relationships with Commercial Interests:** P. J. Turner has received consultancy fees from the UK Departments of Health with respect to primary prevention strategies for food allergy; co-authored guidance on primary prevention for the British Society for Allergy and Clinical Immunology; was co-investigator on the Beating Egg Allergy (BEAT) study (a primary prevention trial for egg allergy) funded by the Ilhan Food Allergy Foundation. D. E. Campbell is a member of the National Allergy Strategy working group to implement primary prevention food allergy strategies (funded by the Australian Federal Government); co-authored guidance from the Australian Society of Clinical Immunology and Allergy with respect to primary prevention; and was chief investigator for the BEAT study, funded by the Ilhan Food Allergy Foundation. R. J. Boyle received consultancy fees by the UK Food Standards Agency to undertake a meta-analysis and systematic review of primary prevention strategies for food allergy. M. E. Levin has no relevant conflicts of interest to declare. M. Schatz discloses no relevant financial relationships.

The mainstay of food allergy management has been allergen avoidance and the provision of rescue medication in the event of accidental reactions. The lack of alternative robust treatment options, together with an increasing prevalence in many countries,^1^ has created a major public health concern. This has stimulated research into the processes underlying the development of food allergy, with the aim of identifying effective prevention strategies. Such strategies may be population based or targeted to an individual, and can be divided into primary prevention (which aim to prevent disease before its onset) and secondary prevention (where early signs are targeted to mitigate or halt disease).^2^

Clinical trials are generally undertaken with significant resources, optimal conditions, and homogeneous participants with limited geographical and environmental variation. Such studies are efficacy trials—assessing the outcome of an intervention under ideal conditions. This is in contrast to effectiveness trials, which are performed under real-life, pragmatic conditions. It is insufficient to demonstrate that an intervention is successful within the confines of a randomized controlled trial (RCT) to ensure that it will be an effective intervention across the population at large.^3^ Population-based interventions need to be assessed not only for level of evidence, applicability, and feasibility before recommendations are made, but they also need to be evaluated once implemented, such that their actual impact, and any unintended consequences (or harm), can be determined.

In this commentary, we discuss the main challenges and risks of using data from efficacy trials to develop public health interventions appropriate to the general population, and explore barriers to the successful implementation of measures to reduce the community burden of food allergy.

## Assessing the Quality of Evidence and Strength of Recommendations

**Figure 1:**
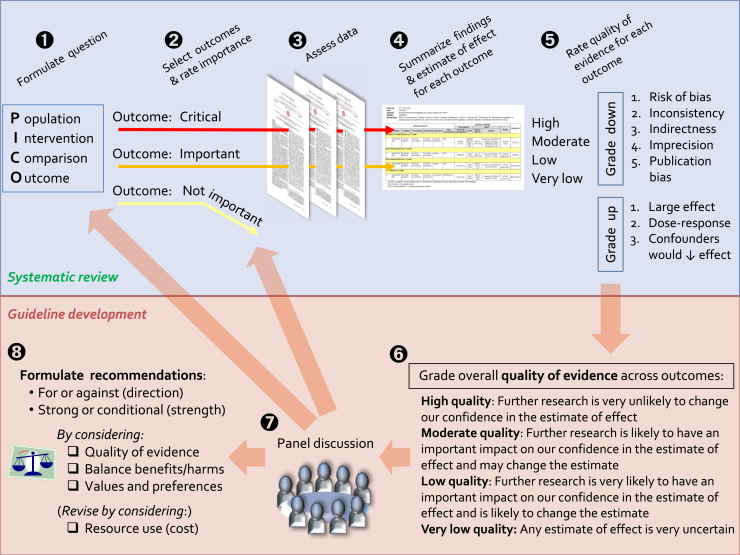
Schematic representation of the GRADE approach for synthesizing evidence and developing recommendations.

Adapted with permission from: Schünemann H, Brożek J, Guyatt G, Oxman A, editors. GRADE handbook for grading quality of evidence and strength of recommendations. Updated October 2013. The GRADE Working Group, 2013. Available from: www.guidelinedevelopment.org/handbook. Accessed January 23, 2018.

The first requirement for any disease prevention program is ensuring the sensible and accurate translation of evidence arising from clinical trials into public health recommendations and policy. This is a complex process that is best approached in a systematic way. The Grading of Recommendations Assessment, Development and Evaluation (GRADE) working group has developed a commonly used tool to evaluate the certainty of findings arising from a systematic review of the evidence,^4^, ^5^ and a separate tool to assist with making recommendations for treatment, diagnosis, or prevention.^6^ These are summarized in Figure 1. In brief, the available evidence for each outcome of interest is first assessed for quality, from very low to high, according to the confidence in the evidence. This may be downgraded because of variety of reasons as outlined in Table I. Once the quality of the evidence has been assessed and considered sufficiently robust to merit consideration for translation, the applicability to the wider population must then be evaluated (Table II). A strong recommendation would be appropriate when most patients (or their families) would want the intervention, where the majority of clinicians agree that the intervention should be offered, and where the recommendations are acceptable as a public health measure to policy makers.^8^

**Table I:** Factors that reduce the confidence in evidence for primary prevention strategies—as applicable to studies assessing the impact of early introduction of allergen into the diet for primary prevention

Factor	Examples
Study limitations	• Lack of blinding of intervention that may bias parents to report reactions, impact on compliance with unblinded intervention, etc • Large loss to follow-up • Failure to conduct intention-to-treat analysis—per protocol analyses tend to distort the data in prevention studies for food allergy, by removing infants who have early allergic reactions due to the intervention in the treatment but not the placebo group • Early termination of study due to apparent benefit^7^
Inconsistent results	• Different estimates of the intervention effect across different studies for the same intervention • Heterogeneity in effect across different populations • Heterogeneity in effect for the same intervention, for different allergens (in the absence of a plausible explanation)
Indirectness of evidence	• Intervention uses a form of allergen that is unlikely to be available or acceptable to other populations • Comparing interventions that use different formulations of the same allergen • Differences in the study population, intervention, or outcome of interest compared with the wider population, eg, extrapolating findings from a study assessing the impact on sensitization to assess the impact on food allergy, rather than using challenge-proven food allergy as the outcome measure
Imprecision	• Study includes relatively few participants or has few events, resulting in wide confidence intervals (this is why many PP studies are undertaken in high-risk populations, to increase the rate of food allergy in the control group)
Publication bias	• Failure to report or journals to publish studies with negative findings

*PP*, Primary prevention.

**Table II:** Applying the evidence to the population

	Individual	At-risk population	Wider population	Health system and public health recommendations
Priority of the problem	High if family history	High	Possibly lower.	Low priority compared with other health conditions
Applicability/generalizability of the evidence	High—where evidence is from studies in high-risk populations		Less applicable to lower risk groups, as the effect of intervention is less	
Benefits vs harms	Benefits likely to outweigh harms, with a smaller NNT to achieve benefit		Benefits may be less likely to outweigh harms, with a larger NNT to achieve benefit and other unseen consequences of intervention becoming more important	
Resource use	Are the out-of-pocket costs relative to the benefits in favor of the intervention?		Does the cost-effectiveness of the intervention favor the intervention?	Is the intervention cost-effective compared with other public health interventions?
Equity			What is the impact on health equity?	
Acceptability	Is the intervention acceptable to the individual, their carers, and health care provider?		Is the intervention acceptable to key stakeholders?	Is the intervention more acceptable than alternatives including health interventions for other diseases?
Feasibility	Is the intervention feasible to the individual, their carers, and health care provider?		Is the intervention feasible to a high-risk population, their carers, and health care provider?	Is the intervention feasible to implement at a public health level?

*NNT*, Number needed to treat.

The GRADE framework has been used by the Allergic Rhinitis and its Impact on Asthma group and working groups within the World Allergy Organization (WAO),^9^ and by WAO to make recommendations regarding the use of probiotics for allergic disease prevention.^10^ However, with respect to food allergy prevention, although there have been several recommendations arising from national specialist organizations, it is not clear that any of these have yet undertaken this robust approach to formulating recommendations to be implemented at a population level.

## Intervention Strategies for Prevention of Food Allergy

Table III summarizes existing synthesized evidence for food allergy prevention interventions of current high interest using the GRADE approach. Although it is beyond the scope of this review to discuss each intervention in detail, there are only a few interventions that currently have sufficient evidence to warrant consideration as a population-based implementation. These include early introduction of peanut and egg to the infant diet,^17^ maternal probiotic supplementation during pregnancy and lactation,^10^ and maternal fish oil supplementation during pregnancy.^11^, ^12^, ^14^ The remainder of the synthesized evidence to date shows low or no evidence when assessed using the GRADE approach,^13^, ^15^, ^16^, ^18^, ^19^ including allergen avoidance during pregnancy and lactation.^11^, ^12^, ^13^ We do not discuss probiotics or fish oil further in this commentary, because the evidence for their effectiveness is of either indirect or low quality (Table III) and neither are widely recommended for food allergy prevention in most current guidelines.

**Table III:** Assessment of synthesized evidence for prevention of food allergy using the GRADE approach

Strategy	Effect size	GRADE quality of evidence	Comments
Antenatal			
• Maternal allergen avoidance	No evidence that maternal allergen avoidance is effective in reducing FA or sensitization^11^, ^12^, ^13^	No evidence	There is increasing evidence that maternal avoidance increases the risk of allergic sensitization and food allergy in offspring
• Maternal fish oil supplementation	Fish oil reduces sensitization to egg: RR 0.55 [95% CI 0.40-0.76]^11^, ^12^, ^14^ Fish oil reduces sensitization to peanut: RR 0.62 [95% CI 0.40-0.96]^11^, ^12^, ^14^	⊗ ⊗ ⊗ ○ Moderate	Evidence downgraded for indirectness: no evidence of the impact on FA outcomes (as opposed to sensitization). However, recommendation is cheap and acceptable
• Maternal probiotics	No evidence^10^, ^11^, ^12^	○ ○ ○ ○ No evidence	Poor study quality, indirect outcome, inconsistent with data related to other outcomes
During lactation			
• Maternal allergen avoidance	No evidence to suggest avoidance is an effective strategy^11^, ^12^, ^13^	○ ○ ○ ○ No evidence	
• Maternal probiotics	No evidence that probiotics influence risk of food allergy^10^, ^11^, ^12^	○ ○ ○ ○ No evidence	
• Maternal fish oil	No evidence^11^, ^12^, ^14^	○ ○ ○ ○ No evidence	Most studies assessed fish oil supplementation during pregnancy ± lactation
Infant feeding			
• Hypoallergenic formula	No consistent evidence that partially or extensively hydrolyzed formula reduces risk^15^	○ ○ ○ ○ No evidence	
• Infant prebiotics	No evidence that prebiotics reduce the risk of atopic disease or food allergy: data sparse^11^, ^12^, ^16^	○ ○ ○ ○ No evidence	
• Infant probiotics	Probiotics may reduce sensitization to cow's milk but not other allergens: RR 0.60 [0.37-0.96]^10^, ^11^, ^12^	⊗ ⊗ ○ ○ Low	These trials used a combination of maternal and infant supplementation interventions; it is unclear as to the relative effects of each of these in isolation. Evidence downgraded for indirectness and imprecision
• Age of introduction of allergenic foods	Introduction of egg from 4 to 6 mo reduces the risk of egg allergy: RR 0.56 [95% CI 0.36-0.87]^17^ Introduction of peanut from 4 to 11 mo reduces the risk of peanut allergy: RR 0.29 [95% CI 0.11-0.74]^17^	⊗ ⊗ ⊗ ○ Moderate ⊗ ⊗ ⊗ ○ Moderate	Reduced because of indirectness of evidence (some studies only recruited infants without any sensitization, thus excluding already-sensitized infants) Evidence downgraded for imprecision and indirectness (control intervention, ie, peanut avoidance to age 5 y is not representative of population norms), but increased due to strength of effect
Other			
• Skin interventions∗	No evidence that skin care impacts on FA or sensitization: RR 0.92 (95% CI 0.58, 1.46) for sIgE to egg >0.35 kU/L^18^ RR 0.45 (95% CI 0.13-1.61) for positive SPT to a food allergen at 12 mo^19^	○ ○ ○ ○ No evidence	

*CI*, Confidence interval; *FA*, food allergy; *GRADE*, Grading of Recommendations Assessment, Development, and Evaluation; *RR*, risk ratio; *SPT*, skin prick test.

∗ Not synthesized data.

## Generalizability of Evidence

Generalizability is a primary limitation of the early allergenic food introduction trials,^14^ particularly for peanut. The evidence that early peanut introduction reduces the risk of peanut allergy is largely derived from a single study, the Learning Early About Peanut (LEAP) study.^20^ In this single-center study, treatment compliance was very high, and the advice given to the control group (complete peanut avoidance until age 5 years) is not consistent with current advice in most countries. This is relevant when attempting to apply the results of LEAP to a general population, where levels of compliance with the active intervention are likely to be lower than they were in the original trial,^21^ and the “no intervention” option is not equivalent to strict avoidance of peanut to age 5 years.^22^ Evidence of efficacy for early introduction of egg comes from a number of RCTs that have been the subject of meta-analysis.^17^ The risk ratio for the impact of early egg introduction on the risk of egg allergy is surprisingly consistent across those trials completed to date, despite differences in intervention, timing of introduction, and the population studies. In contrast, there is significant variation in adverse events according to the nature of the intervention (ie, boiled egg vs raw egg powder). So for egg, there appears to be less of an issue with regard to efficacy outcomes, but a question over generalizability in terms of how the formulation of egg (boiled, raw egg powder, etc.) impacts on the frequency and nature of adverse events.

The impact of any given intervention is closely related to the risk of developing food allergy in the population under study. On the basis of a recent systematic review,^17^ the number needed to treat (NNT) to prevent a single case of egg allergy is relatively high in an unselected population, but lower for infants with early onset moderate-severe eczema—a group at higher risk for egg allergy^23^ (Table IVA). Data for peanut are more sparse, because they are derived in the main from a single trial (LEAP) and because peanut allergy is less common than egg allergy. However, the picture is similar: early introduction in an unselected population requires a relatively large number needed to treat, compared with a population of infants with early onset moderate-severe eczema and/or egg allergy (Table IVB). However, the well-described “prevention paradox” acknowledges that effective population-based interventions are generally more useful than those that target specific at-risk groups—where interventions can achieve large overall health gains for whole populations but may offer only small advantages to each individual.^24^

**Table IV:** Number needed to treat with early introduction of egg and peanut, stratified by risk

Population	Control risk/1000 infants	Intervention risk/1000 infants	Risk difference	95% CI	Number needed to treat
(A) Absolute risk differences for different populations associated with early introduction of egg					
Normal risk∗	54	30	24	7-35	42 (29-143)
High risk†	100	56	44	13-64	23 (16-77)
Very high risk‡	500	280	220	65-320	5 (3-15)
(B) Absolute risk differences for different populations associated with early introduction of peanut					
Normal risk∗	25	7	18	6-22	56 (45-167)
High risk‡	170	49	121	44-151	8 (7-23)

Control risks are estimated from included studies or when relevant from other large population-based studies for populations at different risks of the outcome.^17^

*CI*, Confidence interval.

∗ Risk refers to the unselected population of infants.

† Infants at a high hereditary risk of allergic disease.

‡ Infants with moderate-to-severe eczema.

## Applicability—Variations in Populations and Setting

Food allergy prevention research has largely been conducted in resource-rich settings with a high prevalence of food allergy. This poses considerable challenges when extrapolating these data to other settings,^25^ not just in terms of resources and acceptability (as discussed below) but also in the assessment of the effect. Simply put, the impact of an intervention depends on the local prevalence of food allergy, whether the “proven” intervention is efficacious in the population in question, and if it can be effectively implemented in the setting.

Epidemiological data on food allergy prevalence is limited in many regions,^1^ and confounded by differences in methods of diagnosis and bias due to sampling specialized populations or low response rates. The most rigorous data, based on food challenges in unselected populations, are only available for the USA, Europe, China, Thailand, Australia, and South Africa.^26^ Lower rates of background risk reduce the impact of interventions, increasing the NNT, although there are data to suggest that the prevalence of food allergy in low-middle income countries may be increasing to similar levels as high-income countries,^27^ as these countries undergo modernization and expose the population to more proinflammatory predisposing factors and less rural, protective factors. Primary prevention strategies impact on the processes that lead to sensitization and onward to clinical reactivity, but these same processes are affected by urbanization,^28^ socioeconomic class,^29^ ethnicity,^30^ and migration patterns.^31^, ^32^ These factors all increase the uncertainty of effect when extrapolating data from research to other populations.

## Unintended Consequences of Community-Based Prevention Strategies

Although food allergy prevention is a clear benefit of any given intervention, less is understood about the potential harm that might result from population-based policies. The medium-to long-term impact of early allergen introduction on maternal and child health outcomes has not been fully assessed, and is potentially of greater concern in low-medium income settings. Although beneficial effects have been shown for the prevention of a specific food allergy in the child administered the intervention, it is not clear whether the overall benefit of the intervention outweighs the burden of treatment, and whether there are adverse effects for the child or other household members.

Exclusive breastfeeding during the first 6 months of life (and by definition, delayed introduction of solids and other liquids) is the current World Health Organization advice irrespective of the geographical region. Whilst early food introduction strategies do not promote early cessation of breastfeeding, the duration of exclusive breastfeeding is clearly reduced and there may be a reduction in the overall duration of breastfeeding, although this has not been observed when examined in the context of RCTs to date.^33^, ^34^ The benefits of breastfeeding are clear, with a significant reduction in the risk of infant otitis media, lower respiratory tract infections, gastroenteritis, with possible beneficial effects on child intelligence and adult economic productivity, reduced obesity, and incidence of diabetes.^35^ There is evidence that “partial breastfeeding” (the combination of breastfeeding with other fluids or solids) is less effective at protection against childhood infections than exclusive breastfeeding,^36^ and that partial breastfeeding is high-risk for human immunodeficiency virus (HIV) transmission.^37^ This is likely to be particularly relevant in low-medium income settings such as Africa and Asia, where infant mortality and morbidity from infections remains high, and where a large proportion of the world's population reside. At the same time, background rates of food allergy are lower in these areas, so the population-level benefit of interventions to prevent food allergy is less. In those regions where food allergy is increasing, along with conditions associated with poverty such as HIV and malnutrition, there is a significant challenge in devising infant feeding strategies for atopic children: public health advice to introduce complementary foods early may be ill-advised and an individualized approach may be required.^38^

Concerns have also been raised as to the consequences of displacement of breast milk and weaning foods by high-calorie foods such as peanut and egg. Data from the LEAP,^20^, ^39^ Enquiring About Tolerance,^33^, ^40^ and Beating Egg Allergy^34^, ^41^ studies do not indicate any significant short-term detrimental effects on growth^33^, ^34^, ^39^; however, these studies were conducted in highly selected and motivated families largely from higher socioeconomic backgrounds with higher levels of education, in resource-rich settings. Whether a similar neutral impact would be seen at a population level, in the absence of the monitoring and advice available within a clinical trial, is unclear and of significant concern. A UK government risk assessment also considered toxicological issues as a potential risk—for example, aflatoxin (peanut) or salmonella (egg) exposure to very young infants with early allergen exposure,^22^ which illustrates the need to consider potential risks beyond those which may seem relevant to the allergy community.

Another possible unintended harmful consequence of food allergy prevention through early exposure to allergenic foods is screening. Screening has been proposed as an important step before early introduction.^42^ However, there are considerable logistical risks to the resourcing of such a recommendation, as we have outlined before, which might impact on the benefit of early introduction and cause harm, by delaying the timing of introduction.^43^ It is not inconceivable that parents might be deterred by a screening and introduction process, due to limited financial resources or access to screening/supervised introduction, or out of fear they have missed a crucial window of opportunity to introduce the food. This would result in a paradoxical effect, where overproscriptive guidelines to support early introduction have the opposite effect and increase the risk of food allergy in later life.

## Feasibility, Equity and Resource Use

The most successful examples of population-based intervention include those that require little active participation from the population, such as water fluoridation for the prevention of dental caries and vitamin fortification of staple foods. In both cases, large proportions of the population receive the intervention regardless of risk stratification for disease or even personal knowledge. Other successful interventions where a higher level of active participation is required have achieved large population uptake in many regions. Examples include folate supplementation in pregnancy, neonatal vitamin K administration, and childhood vaccination. Success has been achieved by the incorporation of interventions into standard models of care, with opt-out (rather than opt-in) policies, or by linking participation with the intervention to a desired resource (eg, school enrolment, childcare, or welfare support payments being linked to vaccination).

Strategies that require individual “screening” to assess suitability of an intervention (such as those recommended by National Institute of Allergy and Infectious Diseases^42^ before the introduction of peanut into the infant diet) increase the resource needed for implementation, may delay timing of the intervention, and also exacerbate issues of equity of access and resource. This can also impact on the effectiveness of an intervention: for example, families may have to wait many months for “screening” because of a lack of access or ability to finance health services, which in turn delays the timing of early introduction and therefore its effectiveness. Moreover, with respect to food allergy prevention, the arguments for “screening” currently lack a fundamental evidence base of efficacy and cost-effectiveness.

Resources vary considerably from one country to another, and even within the same country. The substantial disparity in health outcomes between countries of differing socioeconomic status requires attention to those disorders contributing most to the burden of disease in lower-middle income countries. Moreover, in less developed settings, lower public awareness of food allergy and under-awareness of health care providers, together with competing demands on the health system, will result in altered health and prevention priorities for individuals and for governments in these settings.^26^ Acceptability of food allergy primary prevention strategies in these settings may also be reduced.

## Evaluating Prevention Recommendations and Policies: Lessons and Future Directions

An intervention may be based on high-quality evidence that in turn drives a strong recommendation for implementation, can appear applicable and feasible, and yet still not be effective across a population. This can be due to failure of implementation, or unforeseen lack of effectiveness at a population level. Thorough evaluation of population-targeted prevention strategies is therefore crucial in determining real-life effectiveness and in identifying unintended consequences and harms. Public-health recommendations should never be made with a “let's try it and see…” approach: an intervention must have a coherent plan for evaluation included in the implementation plan.^44^

The universal or opt-in models presented above for health prevention (water fluoridation, vitamin fortification of foods, etc.) are unlikely to be applicable to the current proven interventions available for primary prevention of food allergy at a population level. As shown in Table III, high-quality RCT evidence for prevention strategies is currently limited to early introduction of peanut and egg into the diet of infants, with the most marked effects confined to a high risk population. Evidence may emerge of interventions that may be more amenable to passive population-based implementation, but the current proven strategy requires high levels of active participation and effective dissemination of information and education. This does not bode well, as the evidence is that population-based interventions that require high levels of participant participation and population-wide education are in general ineffective: for example, smoking, obesity, and skin cancer prevention interventions have been largely disappointing.

On the other hand, the current evidence for earlier introduction of peanut and egg is also consistent with the existing epidemiological data that delaying these allergens into the infant diet increases the risk of developing allergy to these foods.^11^, ^12^ The original basis for LEAP came from epidemiological comparisons in peanut consumption (and specifically, age of introduction) between Israel and the UK.^20^ Strategies that seek to reverse previous advice to delay introduction (such as that recommended by ASCIA in Australia)^45^ may be more successful at a population level than attempts to actively promote “early” introduction.

Looking specifically at uptake of current and past infant feeding guidelines, the evidence that education and dissemination of feeding recommendations alone impacts on infant feeding practices is poor. Prior to the USA, Europe, the UK, and Australasia changing their infant feeding guidelines around 2008 (in response to emerging data from observational cohort studies that did not suggest a protective effect for the delayed introduction of allergenic food into infants' diet), there was population-based data to suggest that many families did not heed or follow this advice.^46^ Similarly, despite the near-universal recommendation to exclusively breastfeed for at least 4 months in high-income countries, only a relatively small proportion of mothers in these regions achieve this target. In the UK, 30% of infants had already had solids introduced into their diet by age 4 months, 75% by 5 months, and 94% by 6 months.^47^ In Australia, only 39% of infants were exclusively breastfed after 3 months of age in 2010.^48^

Specific recommendations for the prevention of peanut allergy have now been released in at least 2 regions, the USA^42^ and Australia.^45^ They vary considerably in their approach: one with a specific emphasis on screening for suitability (a high resource model), the other minimizing the role of screening and aiming for broader uptake. This creates an opportunity to examine how these different approaches will be taken up and accepted by the population at large, and whether they will be effective in reducing rates of peanut allergy. Now is the time to put in place robust systems for measuring uptake and outcome in these regions, so we can make clear assessments about the effectiveness of each approach and in turn inform implementation recommendations for other regions.

## Summary

Public health strategies that require active participation from the community require individuals and families to not only know about changes in recommendations, but to knowingly alter behavior. This can be particularly difficult where infant feeding is concerned. There is little existing evidence that it is possible to achieve a widespread behavioral change in a complex area such as infant feeding, where so many cultural, resource, and emotional factors are at play.

Families vary in their preferences and perception of risk. Some may prioritize the introduction of food allergens early during weaning because of family history or a specific concern over the potential for food allergy. For others, the same specific drivers may increase anxiety over the potential for an allergic reaction, and interfere with their willingness to undertake early introduction. Such individual preferences will differ from health care professionals, who might be concerned as to their advice resulting in an allergic reaction, and policy makers who need to balance the risks of allergic reactions occurring in the community with the benefits on reducing the risk of food allergy.

It is insufficient for population-based interventions to be based on the highest level of evidence; they also need to be generalizable, simple, cheap, doable, and have the ability to be evaluated after implementation. There are few interventions at preventing food allergy that pass the high evidence bar, although this will hopefully change as more evidence is generated from studies of high quality in heterogeneous populations and across regions. Until then, we remain in a transitional phase: it therefore behooves us to exercise appropriate caution when developing and implementing strategies aimed at primary prevention of food allergy, at a population level.
